# Clinical characteristics of pediatric patients with confirmed SARS-CoV-2 infection who followed rigorous measures during two years of the COVID-19 pandemic in a hospital in Mexico

**DOI:** 10.3389/fped.2023.1150738

**Published:** 2023-06-27

**Authors:** Ana Carolina Ramírez-Cázares, Yodira Guadalupe Hernández-Ruíz, César Adrián Martínez-Longoria, César Eduardo Tamez-Gómez, Obed Medina-Macías, Roberto Guadalupe Treviño-Montalvo

**Affiliations:** ^1^ Vicerrectoría de Ciencias de la Salud, Escuela de Medicina, Universidad de Monterrey, San Pedro Garza García, Mexico; ^2^ Pediatric Department, Hospital Clínica Nova, San Nicolas de los Garza, Mexico; ^3^ Research Department, Hospital Clínica Nova, San Nicolas de los Garza, Mexico

**Keywords:** children, pediatrics, SARS-CoV-2, epidemiology, COVID-19

## Abstract

**Background:**

COVID-19 infections have been described as asymptomatic or mild, with a low incidence of severe cases in children and adolescents who followed the basic hygiene measures. The objective of this study was to describe the clinical and epidemiological characteristics of our pediatric population during four waves of the COVID-19 pandemic from a private hospital.

**Methods:**

A retrospective observational study in patients under 15 years old with confirmed SARS-CoV-2 infection by real-time reverse transcription-polymerase chain reaction (RT-PCR) test from April 1, 2020 to April 30, 2022. Demographic, clinical, and therapy variables were examined, and the Chi-square test was used for comparisons.

**Results:**

From 5,870 RT-PCR taken through the first two years of the pandemic, 1,371 tested positive, obtaining a positivity rate of 23.37%. Patients' median (IQR) age was 9.0 (7.0) years, and most were male (*n* = 705, 51.4%). The primary comorbidities were rhinitis (*n* = 239, 17.4%) and asthma (*n* = 172, 12.5%). Most cases were scholars (*n* = 568, 41.4%) during the fourth COVID-19 wave (*n* = 831, 60.6%). Almost all cases (88.2%) reported prior exposure to SARS-CoV-2-infected households. Six percent (*n* = 82) of the patients reported being vaccinated against SARS-CoV-2. Most participants (89.3%) received outpatient care, and 0.6% required hospitalization. Nine (0.6%) patients were diagnosed with Multisystemic Inflammatory Syndrome in Children (MIS-C). The second COVID-19 wave reported a higher frequency of anosmia and dysgeusia; the third wave reported fever and malaise, and the fourth wave reported cough, odynophagia, and vomiting (*p* < 0.05). The second wave reported no treatment (*n* = 23, 15.3%), while the third and fourth waves reported outpatient care and hospitalization (*n* = 367, 95.1%; and *n* = 4, 1.0%, respectively) (*p *= <0.001). Reinfection cases were frequent during the second wave (*n* = 8, 5.3%) (*p*=<0.001). Rhinorrhea, vomiting, and diarrhea were reported mainly by infants; fever by preschoolers; abdominal pain by scholars; and headache, odynophagia, anosmia, dysgeusia, myalgia, arthralgia, and malaise by adolescents (*p* < 0.05). Neither treatment nor reinfection showed age-related differences (*p *= 0.496 and *p *= 0.224, respectively).

**Conclusion:**

The study demonstrated a lower positive rate for SARS-CoV-2 in our hospital'The majority of cases in our study were outpatients who reported a mild infection with a favorable evolution based on symptomatic treatment.

## 1. Introduction

Two years have passed since the World Health Organization (OMS) was forced to recognize the novel coronavirus disease (COVID-19) as a pandemic (1). In the beginning, the most affected population was adults. A systematic review of the literature on COVID-19 patients from China, Italy, Spain, and the United States, reported that pediatric cases were a minority (<2%) (2). Until April 30th, 2022, Mexico reported 5,761,682 confirmed cases, of which 281,365 (4.8%) were under 16 years of age (3, 4).

To minimize exposure to the virus, the Mexican government enforced a lockdown, shutting down businesses, schools, and non-essential services so that people would stay home (5). People were encouraged to follow basic hygiene measures, social distancing, and work from home (6, 7). The government used a four-tired traffic light monitoring system, based on the epidemiological risks of contagion for SARS-CoV-2, to inform citizens about the use of public spaces (8).

As the pandemic continued, pediatric COVID-19 reported asymptomatic or mild symptoms, with a lower risk of hospitalization and complications (9, 10). Two main factors could make the SARS-CoV-2 diagnosis relatively challenging for this group. The first factor is the non-specific clinical features, which may lead to misleading the cause with other viral pathogens. The second factor is the increase in suspected cases with no diagnostic tests available.

The state of Nuevo Leon, México, reported 319,488 pediatric confirmed cases until April 30th, 2022 (4). Hospital Clinica Nova (HCN), a private hospital in this state, had 44,186 beneficiaries, of which 10,673 were under 16 years of age. This hospital only provides attention to a company's employees and their families; hence the pediatric population had a close follow-up. To avoid COVID-19-positive cases, this company implemented a safety protocol among its workers, used diverse strategies to spread information about the pandemic, and established COVID-19 attention units at HCN.

Nevertheless, the impact of these measures was unknown. This study aimed to describe the clinical and epidemiological characteristics of our pediatric population during four waves of the COVID-19 pandemic. As a second objective, we described the contingency measures that took place in HCN to reduce the virus transmission.

## 2. Material and methods

### 2.1. Design

This retrospective observational study included patients under 16 years of age with confirmed SARS-CoV-2 infection by real-time reverse transcription-polymerase chain reaction (RT-PCR) test at HCN from April 1st, 2020 to April 30th, 2022.

The study received approval from the local Institutional Review Board (Ref.: 10092021-CN-1a-CI). It followed the STROBE guidelines and the Code of Ethics of the World Medical Association (Declaration of Helsinki) for experiments involving humans (11). The use of a consent form did not apply due to the retrospective nature of this study.

### 2.2. COVID-protocol attention in the hospital

The HCN implemented diverse strategies to guide patients through the health crisis; this could be divided into two main approaches: social media/technology use and exclusive attention units at the hospital. The first approach used telemedicine instead of face-to-face appointments, webinars, and social networks to communicate updates on the COVID-19 outbreak. Besides, HCN established a service center called “COVID-Line” for counseling on suspected cases and follow-ups, so patients did not need to go to the hospital for medical advice. The second approach consisted of an exclusive area for respiratory/febrile cases to evaluate the severity of the clinical manifestations, whether a COVID-19 test was necessary, and decide the general management.

Indications for testing included at least one of the following: fever, respiratory/gastrointestinal symptoms, asthenia, anosmia, hypogeusia, or a positive close contact. Also, RT-PCR was mandatory in patients who had surgery. All non-severe cases received outpatient care, which consisted of symptomatic management with acetaminophen, Non-Steroidal Anti-Inflammatory Drugs (NSAIDs), antihistamines, and probiotics if needed.

### 2.3. Data collection

The department of epidemiology collected data on patients who attended to discard a SARS-CoV-2 infection. This database helped us to search for patients according to the study's inclusion criteria. We used the Mexican National database to calculate the national and statewide positivity rate of the RT-PCR for SARS-CoV-2 compare them with the HCN positivity rate (4). The inclusion criteria were individuals of both genders under 16 years of age who were HCN beneficiaries with confirmed SARS-CoV-2 infection by RT-PCR from April 2020 to April 2022. The exclusion criteria were a negative RT-PCR test for SARS-CoV-2 or another diagnostic test for SARS-CoV-2.

The variables we analyzed were: age, gender, comorbidities (e.g., obesity, overweight, asthma, rhinitis), infected household members confirmed by RT-PCR test, date when the RT-PCR test was taken, immunization schedule, clinical manifestation during the SARS-CoV-2 infection, treatment (e.g., no treatment at all, outpatient care or hospitalization), complications and reinfection.

We stratified the sample into two primary analyses: COVID-19 waves and age groups. COVID-19 waves were considered as follows: the first wave from April 1st to August 31st, 2020; the second from September 1st, 2020 to April 30th, 2021; the third from May 1st to November 30th, 2021; and the fourth from December 1st, 2021 to April 30th, 2022. The age groups were considered as follows: infants (0–1 years), preschoolers (2–5 years), scholars (6–11 years), and adolescents (12–15 years).

### 2.4. Statistical analysis

The researchers reviewed the quality control and anonymization of the database. We used descriptive statistics for the categorical data, such as frequencies and percentages. We used median and interquartile range (IQR) for quantitative variables since they showed no normal distribution. The Chi-square test was used to compare characteristics between groups. The data were processed on the statistical program SPSS, version 25. The analysis was two-tailed, and a *p*-value <0.05 was considered statistically significant.

## 3. Results

From April 1st, 2020 to April 30th, 2022, 5,870 pediatric patients underwent RT-PCR testing, and 1,371 tested positive for SARS-CoV-2. The age group with more SARS-CoV-2 cases was the scholars (*n* = 568, 41.4%), and the COVID-19 wave with more infections was the fourth wave (*n* = 831, 60.6%).

### 3.1. General results

The patients' median (IQR) age was 9.0 (7.0) years, and most participants were male (*n* = 705, 51.4%). The comorbidities reported were: rhinitis (*n* = 239, 17.4%), asthma (*n* = 172, 12.5%), and obesity (*n* = 82, 6.0%). Twelve hundred nine (88.2%) patients reported a household infection; the rest (*n* = 162, 11.8%) was considered a symptomatic index case in their household.

Ten-point one percent (*n* = 138) of the participants reported an asymptomatic infection. Fever was reported in 52.9% (*n* = 725) of the patients. Cough, rhinorrhea, and odynophagia were classified as respiratory symptoms; nausea, abdominal pain, vomiting, and diarrhea as gastrointestinal symptoms. Most patients reported respiratory symptoms (*n* = 1,041, 75.9%), where cough was the most frequent (*n* = 707, 51.6%). Only 223 (16.3%) patients reported gastrointestinal symptoms.

Of all the reported pediatric cases in HCN, 10.1% (*n* = 139) received no treatment. Around 1,224 (89.3%) received symptomatic measures as outpatients; three received unproven therapies (e.g., ivermectin) from an external source. Only eight (0.6%) patients required hospital admission. Of those eight patients: three presented pneumonias, and only one developed severe manifestation, demanding high-flow ventilation management and Remdesivir; two presented another non-COVID-related diagnosis, but an RT-PCR was performed as part of the admittance protocol; one patient was already being treated for chronic renal failure when tested positive for SARS-CoV-2; the last two patients were diagnosed on the preoperative testing for appendicitis surgery.

Twenty-one (1.5%) patients reported SARS-CoV-2 reinfection. The median (IQR) days between the two RT-PCR tests was 207 (202). Nine (0.6%) patients were diagnosed with Multisystemic Inflammatory Syndrome in Children (MIS-C), according to the Centers for Disease Control and Prevention criteria (12). All patients received an immunoglobulin infusion as the initial therapy; only four required dexamethasone as second-line treatment. To date, all patients have reported favorable evolution.

Six percent (*n* = 182) of the patients reported prior immunization against SARS-CoV-2, but only 3.2% (*n* = 44) completed the vaccination schedule. A total of 1,098 (80.1%) patients denied being vaccinated against SARS-CoV-2, and 191 (13.9%) patients' immunization status was unknown. All patients reported receiving the Pfizer-BioNTech (BNT162b2) vaccine.

### 3.2. COVID-19 waves

**Figure 1:**
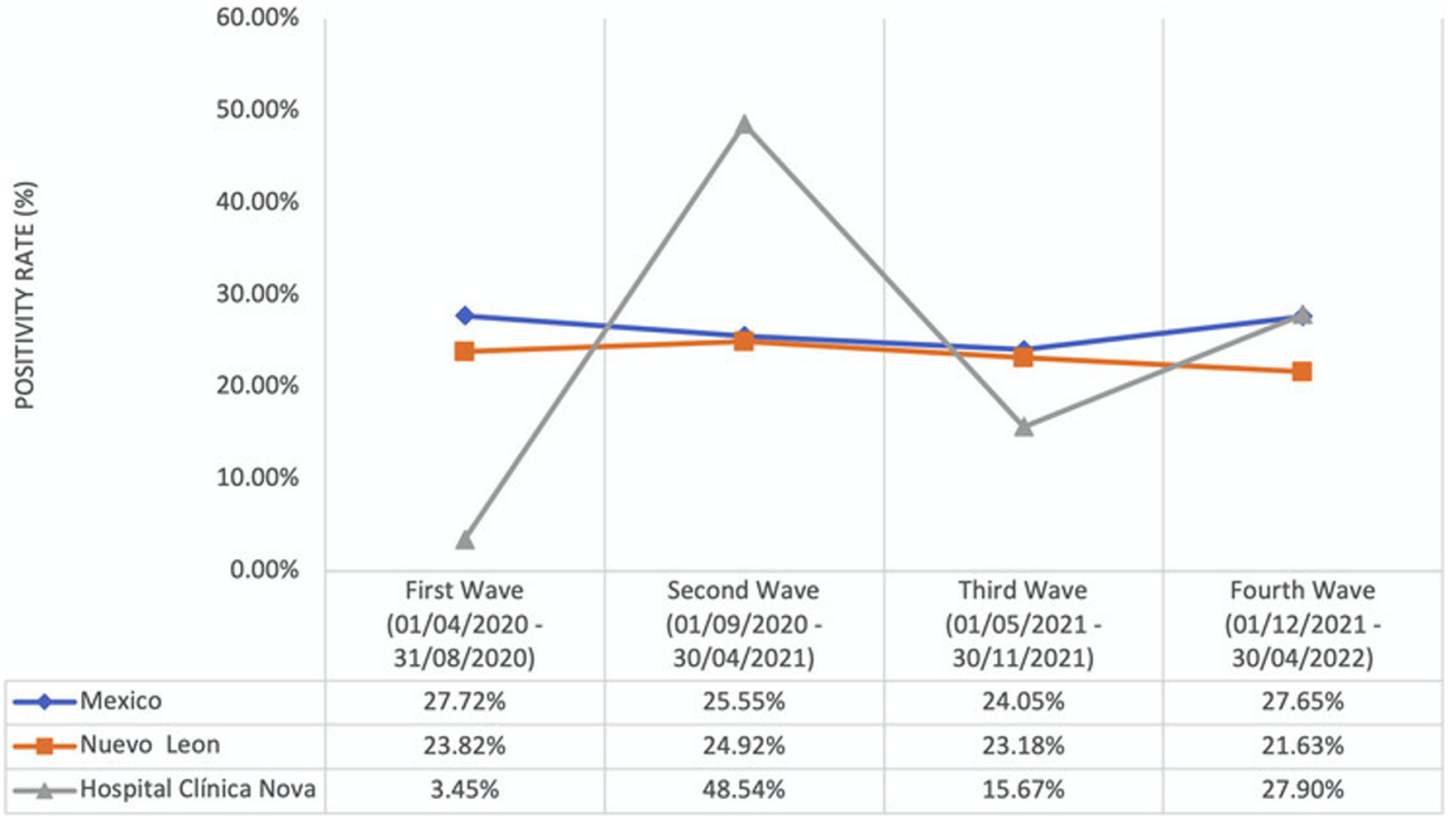
National, statal, and HCN positivity rates of RT-PCR for SARS-CoV-2 according to the four COVID-19 waves. Graph correlating the positivity rates on RT-PCR for SARS-CoV-2. HCN reported the lowest positivity rates during the first two years of the pandemic, except during the second wave. The peak during the second wave was attributed to HCN testing children exclusively after reporting persistent symptoms.

The Mexican National database registered 289,875 RT-PCR for SARS-CoV-2 in the pediatric population, from which 74,678 tested positive, obtaining a positivity rate of 25.76%. The state of Nuevo León reported 3,792 positive tests out of 16,095 RT-PCR, obtaining a positivity rate of 23.56%. At HCN, 1,371 of 5,867 RT-PCR tested positive, obtaining a positivity rate of 23.37%. Figure 1 shows the national, statal, and HCN positivity rates according to the four COVID-19 waves.

The first wave reported only four (0.3%) infected patients; due to the shortage of patients, it was excluded from further data analysis. During the second, third, and fourth waves, 150 (10.9%), 386 (28.2%), and 831 (60.6%) patients were infected, respectively. The second wave reported adolescents as the most affected group (*n* = 77, 51.3%), and the third and fourth reported scholars (*n* = 154, and *n* = 363, respectively; 39.9% and 43.7%, respectively) (*p* < 0.001). There was no significant difference between genders (*p *= 0.137).

During the fourth wave, most patients reported having one or more comorbidities (*n* = 297, 35.7%) (*p *= 0.039). The most frequent conditions were rhinitis [*n* = 178, 21.4% (*p* < 0.001)] and asthma [*n* = 119, 14.3%, (*p *= 0.032)]. An infected household was most common during the fourth wave [*n* = 750, 90.3%, (*p *= 0.025)]. Vaccination status was significantly different among the waves: unvaccinated (*n* = 590, 71.0%), vaccinated (*n* = 76, 9.1%), and unknown status (*n* = 165, 19.9%) were frequently reported during the fourth wave (*p* < 0.001). Table 1 shows the demographic characteristics and medical history according to COVID-19 waves.

**Table 1:** Demographic characteristics and medical history according to COVID-19 waves.

Variable	COVID-19 waves				
	First (*n* = 4)	Second (*n* = 150)	Third (*n* = 386)	Fourth (*n* = 831)	*p*-Value*
Gender					
Male	4 (100%)	68 (45.3%)	211 (54.7%)	421 (50.8%)	0.137
Female	0 (0.0%)	82 (54.7%)	175 (45.3%)	409 (49.2%)	
Age					
Infants (0–1 years)	0 (0.0%)	10 (6.7%)	29 (7.5%)	66 (7.9%)	<0.001
Preschoolers (2–5 years)	0 (0.0%)	14 (9.3%)	94 (24.4%)	168 (20.2%)	
Scholars (6–11 years)	2 (0.0%)	2 (0.0%)	2 (0.0%)	2 (0.0%)	
Adolescents (12–15 years)	77 (51.3%)	109 (28.2%)	234 (28.2%)	77 (51.3%)	
Medical history					
No previous comorbidities	3 (75.0%)	103 (68.7%)	276 (71.5%)	534 (64.3%)	0.039
Previous comorbidities	1 (25.0%)	47 (31.3%)	110 (28.5%)	297 (35.7%)	
Overweight	0 (0.0%)	5 (3.3%)	10 (2.6%)	30 (3.6%)	0.650
Obesity	0 (0.0%)	13 (8.7%)	20 (5.2%)	49 (5.9%)	0.306
Asthma	1 (25.0%)	12 (8.0%)	40 (10.4%)	119 (14.3%)	0.032
Rhinitis	0 (0.0%)	18 (12.0%)	43 (11.1%)	178 (21.4%)	<0.001
Previous contact with a family member with COVID-19					
Positive contact	1 (25.0%)	129 (86.0%)	329 (85.2%)	750 (90.3%)	0.025
COVID-19 vaccine status					
Unvaccinated	4 (100%)	4 (100%)	4 (100%)	4 (100%)	<0.001
Vaccinated	0 (0.0%)	0 (0.0%)	0 (0.0%)	0 (0.0%)	
Unknown	0 (0.0%)	0 (0.0%)	0 (0.0%)	0 (0.0%)	

Data are presented as frequencies (percentages). Chi-square was performed for comparisons only among the second, third and fourth COVID-19 waves.

*Comparison between the initial and final measure; A *p*-value ≤0.05 was considered statistically significant.

The second wave reported the majority of asymptomatic cases (*n* = 27, 18.0%) (*p* < 0.001). Anosmia [*n* = 16, 10.7% (*p* < 0.001)] and dysgeusia [*n* = 13, 8.7% (*p* < 0.001)] were predominant during the second wave; fever [*n* = 239, 61.9% (*p* < 0.001)] and malaise [*n* = 75, 19.4% (*p *= 0.005)] during the third wave. From respiratory symptoms, only cough [*n* = 463, 57.5% (*p *= 0.001)] and odynophagia [*n* = 315, 37.9% (*p* < 0.001)] reported a significant difference during the fourth wave. None of the gastrointestinal symptoms reported a significant difference, except for vomiting, which was most frequent during the fourth wave [*n* = 73, 8.8% (*p *= 0.001)].

Treatments reported a significant difference during the pandemic (*p* < 0.001). No treatment was commonly reported during the second wave (*n* = 23, 15.3%), and outpatient care and hospitalization during the third and fourth wave (*n* = 367, 95.1%; and *n* = 4, 1.0%, respectively). Reinfection cases were frequent during the second wave (*n* = 8, 5.3%) (*p* < 0.001). Table 2 shows the clinical manifestations according to the COVID-19 waves.

**Table 2:** Clinical manifestations according to the COVID-19 wave.

Variable	COVID-19 waves				
	First (*n* = 4)	Second (*n* = 150)	Third (*n* = 386)	Fourth (*n* = 831)	*p*-Value*
Clinical presentation					
Asymptomatic	0 (0.0%)	27 (18.0%)	13 (3.4%)	98 (11.8%)	<0.001
Symptomatic	4 (100%)	123 (82.0%)	373 (96.6%)	733 (88.2%)	
General manifestations					
Fever	3 (75.0%)	46 (30.7%)	239 (61.9%)	437 (52.6%)	<0.001
Headache**	3 (75.0%)	51 (34.0%)	137 (35.5%)	273 (32.9%)	0.661
Anosmia	0 (0.0%)	16 (10.7%)	17 (4.4%)	1 (0.1%)	<0.001
Dysgeusia	0 (0.0%)	13 (8.7%)	7 (1.8%)	3 (0.4%)	<0.001
Myalgia	0 (0.0%)	9 (6.0%)	17 (4.4%)	38 (4.6%)	0.714
Arthralgia	0 (0.0%)	1 (0.7%)	9 (2.3%)	18 (2.2%)	0.441
Malaise	0 (0.0%)	12 (8.0%)	75 (19.4%)	149 (17.9%)	0.005
Respiratory symptoms					
Cough	1 (25.0%)	63 (42.0%)	180 (46.6%)	463 (55.7%)	0.001
Odynophagia	1 (25.0%)	28 (18.7%)	112 (29.0%)	315 (37.9%)	<0.001
Rhinorrhea	2 (50.0%)	81 (54.0%)	167 (43.3%)	405 (48.7%)	0.055
Gastrointestinal symptoms					
Nausea	0 (0.0%)	5 (3.3%)	12 (3.1%)	37 (4.5%)	0.491
Abdominal pain	0 (0.0%)	4 (2.7%)	24 (6.2%)	51 (6.1%)	0.223
Vomiting	0 (0.0%)	3 (2.0%)	16 (4.1%)	73 (8.8%)	0.001
Diarrhea	0 (0.0%)	7 (4.7%)	25 (6.5%)	40 (4.8%)	0.453
Treatment					
No treatment	1 (25.0%)	23 (15.3%)	15 (3.9%)	100 (12.0%)	<0.001
Outpatient care/symptomatic treatment	3 (75.0%)	126 (84.0%)	367 (95.1%)	728 (87.6%)	
Hospitalization	0 (0.0%)	1 (0.7%)	4 (1.0%)	3 (0.4%)	
Reinfection					
Positive reinfection	1 (25.0%)	8 (5.3%)	8 (2.1%)	4 (0.5%)	<0.001

Data are presented as frequencies (percentages). Chi-square was performed for comparisons only among the second, third and fourth COVID-19 waves.

*Comparison between the initial and final measure; A *p*-value ≤0.05 was considered statistically significant.

**Irritability was considered in children under 5 years of age, and headache for 5 years and older.

### 3.3. COVID-19, according to age groups

The scholars were reported as the most affected group (*n* = 568, 41.4%), followed by adolescents (*n* = 422, 30.8%) and preschoolers (*n* = 276, 20.1%). Adolescents were predominantly affected during the second wave (*n* = 77, 51.3%), preschoolers during the third wave (*n* = 94, 24.4%), and scholars during the fourth wave (*n* = 363, 43.7%) (*p* < 0.001). There was no significant difference between genders (*p *= 0.575).

Nearly all infants notified no comorbidities (*n* = 98, 93.3%) (*p* < 0.001). Adolescents reported overweight [*n* = 20, 4.7% (*p *= 0.004)], obesity [*n* = 46, 10.9% (*p* < 0.001)], and rhinitis [*n* = 97, 23.0% (*p* < 0.001)]. Asthma was commonly reported by scholars [*n* = 88, 15.5% (*p* < 0.001)]. There was no significant difference between prior infected households and the age groups (*p *= 0.680). Most toddlers (*n* = 105, 100%) and preschoolers (*n* = 261, 94.6%) reported being unvaccinated. Adolescents mainly reported a vaccinated (*n* = 52, 12.3%) or unknown immunization status (*n* = 77, 18.2%) (*p* < 0.001). Table 3 shows the demographic characteristics and medical history according to age groups.

**Table 3:** Demographic characteristics and medical history according to age groups.

Variable	Age groups				
	0–1 years old (*n* = 105)	2–5 years old (*n* = 276)	6–11 years old (*n* = 568)	12–16 years old (*n* = 422)	*p*-Value*
Gender					
Male	49 (46.7%)	138 (50.0%)	265 (46.7%)	214 (50.7%)	0.575
Female	56 (53.3%)	138 (50.0%)	303 (53.3%)	208 (49.3%)	
Medical history					
No previous comorbidities	98 (93.3%)	216 (78.3%)	360 (63.4%)	242 (57.3%)	<0.001
Previous comorbidities	7 (6.7%)	60 (21.7%)	208 (36.6%)	180 (42.7%)	
Overweight	1 (1.0%)	1 (0.4%)	23 (4.0%)	20 (4.7%)	0.004
Obesity	0 (0.0%)	4 (1.4%)	32 (5.6%)	46 (10.9%)	<0.001
Asthma	1 (1.0%)	24 (8.7%)	88 (15.5%)	59 (14.0%)	
Rhinitis	2 (1.9%)	30 (10.9%)	110 (19.4%)	97 (23.0%)	
COVID-19 wave					
First	0 (0.0%)	0 (0.0%)	2 (0.4%)	2 (0.5%)	<0.001
Second	10 (9.5%)	14 (5.0%)	49 (8.6%)	77 (18.2%)	
Third	29 (27.6%)	94 (34.1%)	154 (27.1%)	109 (25.8%)	
Fourth	66 (62.9%)	168 (60.9%)	363 (63.9%)	234 (55.5%)	
COVID-19 vaccine status					
Unvaccinated	105 (100%)	261 (94.6%)	439 (77.3%)	293 (69.4%)	<0.001
Vaccinated	0 (0.0%)	2 (0.7%)	28 (4.9%)	52 (12.3%)	
Unknown	0 (0.0%)	13 (4.7%)	101 (17.8%)	77 (18.2%)	
Previous contact with a family member with COVID-19					
Positive contact	94 (89.5%)	247 (89.5%)	502 (88.4%)	366 (86.7%)	0.680

Data are presented as frequencies (percentages). Chi-square was performed for comparisons.

*Comparison between the initial and final measure; A *p*-value ≤0.05 was considered statistically significant.

The clinical presentation reported no significant difference among the age groups (*p *= 0.174). Rhinorrhea [*n* = 70, 66.7% (*p* < 0.001)], vomiting [*n* = 12, 11.4% (*p *= 0.006)], and diarrhea [*n* = 17, 16.2% (*p* < 0.001)] were mostly shown in infants. Fever was commonly reported in preschoolers [*n* = 171, 62.0% (*p* < 0.001)]. Abdominal pain was most frequent in scholars [*n* = 48, 8.5%, (*p *= 0.001)]. Headache [*n* = 182, 43.1%(*p* < 0.001)], odynophagia [*n* = 184, 43.6%(*p *= 0.001)], anosmia [*n* = 27, 6.4% (*p* < 0.001)], dysgeusia [*n* = 18, 4.3% (*p* < 0.001)], myalgia [*n* = 29, 6.9% (*p *= 0.002)], arthralgia [*n* = 17, 4.0% (*p *= 0.004)], and malaise [*n* = 104, 24.6% (*p* < 0.001)] were more frequently reported by adolescents. Neither treatment nor reinfection cases were significantly different among the age groups. Table 4 shows the clinical manifestations according to age groups.

**Table 4:** Clinical manifestations according to the age group.

Variable	Age groups				
	0–1 years old (*n* = 105)	2–5 years old (*n* = 276)	6–11 years old (*n* = 568)	12–16 years old (*n* = 422)	*p*-Value*
Clinical presentation					
Symptomatic	6 (5.7%)	25 (9.1%)	55 (9.7%)	52 (12.3%)	0.174
Asymptomatic	99 (94.3%)	251 (90.9%)	513 (90.3%)	370 (87.7%)	
General manifestations					
Fever	65 (61.9%)	171 (62.0%)	305 (53.7%)	184 (43.6%)	<0.001
Headache**	4 (3.8%)	47 (17.0%)	231 (40.7%)	182 (43.1%)	<0.001
Anosmia	0 (0.0%)	0 (0.0%)	7 (1.2%)	27 (6.4%)	<0.001
Dysgeusia	0 (0.0%)	0 (0.0%)	5 (0.9%)	18 (4.3%)	<0.001
Myalgia	0 (0.0%)	5 (1.8%)	30 (5.3%)	29 (6.9%)	0.002
Arthralgia	0 (0.0%)	2 (0.7%)	9 (1.6%)	17 (4.0%)	0.004
Malaise	6 (5.7%)	19 (6.9%)	107 (18.9%)	104 (24.6%)	<0.001
Respiratory symptoms					
Cough	62 (59.0%)	141 (51.1%)	293 (51.6%)	211 (50.0%)	0.425
Odynophagia	8 (7.6%)	63 (22.8%)	201 (35.4%)	184 (43.6%)	<0.001
Rhinorrhea	70 (66.7%)	144 (52.2%)	258 (45.4%)	183 (43.4%)	
Gastrointestinal symptoms					
Nausea	5 (4.8%)	4 (1.4%)	27 (4.8%)	18 (4.3)	0.120
Abdominal pain	0 (0.0%)	14 (5.1%)	48 (8.5%)	17 (4.0%)	0.001
Vomiting	12 (11.4%)	24 (8.7%)	41 (7.2%)	15 (3.6%)	0.006
Diarrhea	17 (16.2%)	13 (4.7%)	27 (4.8%)	15 (3.6%)	<0.001
Treatment					
No treatment	6 (5.7%)	26 (9.4%)	55 (9.7%)	52 (12.3%)	0.496
Outpatient care/symptomatic treatment	98 (93.3%)	249 (90.2%)	510 (89.8%)	367 (87.6%)	
Hospitalization	1 (1.0%)	1 (0.4%)	3 (0.5%)	3 (0.7%)	
Reinfection					
Positive reinfection	3 (2.9%)	1 (0.4%)	11 (1.9%)	6 (1.4%)	0.224

Data are presented as frequencies (percentages). Chi-square was performed for comparisons.

*Comparison between the initial and final measure; A *p*-value ≤0.05 was considered statistically significant.

**Irritability was considered in children under 5 years of age, and headache for 5 years and older.

## 4. Discussion

Our primary objective was to describe the clinical and epidemiological features of COVID-19 pediatric patients at HCN, and secondly, to describe the contingency measures that took place in HCN during the four waves of the COVID-19 pandemic. It's important to remark that HCN only provides attention to a company's employees and their families; hence the pediatric population had a close outpatient follow-up. The majority of positive cases emerged during the fourth COVID-19 wave, where infants were more frequently infected. Most patients showed an asymptomatic or mild infection with a favorable evolution based on symptomatic treatment.

We compared the national, statal, and institutional positivity rates on RT-PCR for SARS-CoV-2. HCN reported the lowest positivity rates during the first two years of the pandemic, except during the second wave (4). The inconsistency could be attributed to the belief that children were less frequently infected than adults, leading HCN to test them exclusively after reporting persistent symptoms (13). On the other hand, our implemented strategy based on educational strategies and closer patient follow-up might explain the lower rates in the next waves.

At the beginning of the pandemic, household contacts were assumed to be the primary source of contagion (14–17). Nevertheless, children have been considered partially responsible for the spread of infectious diseases on account of frequently being asymptomatic carriers (18). During the first two years of the pandemic, 11.8% of our sample was the index symptomatic case in the household. On the contrary, a prospective study by Soriano-Arandres et al. and a systematic review and meta-analysis by Chen et al. mention pediatric index case rates as 7.7% and 10.3%, respectively. Our higher incidence may be related to the fact that we tested every child despite having low-grade symptoms or a non-COVID-19-related primary diagnosis, unlike at the beginning of the pandemic when children were hardly tested (18, 19).

Pediatric COVID-19 cases increased over time. In our sample, infected patients during the fourth wave two-folded those in the third wave (60.6%, and 28.2%, respectively). The Delta SARS-CoV-2 variant triggered the third wave in Mexico. Meanwhile, the Omicron variant emerged by the end of 2021 to cause the fourth wave, reporting the highest number of infected patients in our country. A crucial Omicron characteristic that justifies this phenomenon is its transmissibility, which is 3,31-fold higher than the Delta variant (20). Other reasons for the accelerated increase in pediatric COVID-19 cases during the fourth wave were the relaxation of protective measures, social distancing, and increased social and school activities (21).

Different COVID-19 waves prompted an upgrade in Nuevo Leon's hygiene protocols. During most of the first and second waves, the four-tired traffic light denoted the highest level of risk; it encouraged high-risk populations to visit public spaces under exceptional circumstances. Non-essential locations remained closed or reduced attendance capacity. During the third wave, the vaccination campaign against SARS-COV-2 began; it was divided by age category, beginning with the older population. During the third wave, face-to-face class limitations shifted. In May 2021, middle-higher and higher educational levels could voluntarily return to school; preschool, primary, and secondary levels returned by the end of August 2021. This wave allowed individuals to visit more places as long as they followed the hygiene measures established by the government. However, crowded events were still restricted. The fourth wave reported either an intermediate or low risk of contagion for SARS-CoV-2 in public spaces, and there were no establishments with capacity restrictions. Face mask use in open spaces was optional but obligatory in closed spaces (22).

On the other hand, the transition back to school was challenging. The government allowed higher levels of education to go back to in-person classes before reopening the lower levels. All educational centers had to adhere to the following measures: limit the number of students per classroom, maintain an appropriate distance between students and teachers, ensure that every room has adequate ventilation, give students the option to study at home or go to school, and to have a constant supervision of suspicious symptoms. Masks were mandatory in all schools at all times. The permitted student capacity in classrooms progressively increased over time. By the beginning of 2022, all educational levels will have returned to the school at full classroom capacity. Sanitary measures were required to be observed (22).

The population in this study demonstrated confirmed COVID-19 cases being slightly higher in males (51.4%), similar to other studies such as Martínez-García et al. (59.3%), Olivar-López et al. (53%), Nachega et al. (52.4%), Aragón-Nogales et al. (55.2%), Niño-Serna et al. (54%) (23–27). Contrary to what was described in the literature, we found scholars as the most infected group. A few studies proved that children older than twelve years had a higher frequency of testing positive for COVID-19 (13, 15, 27). Martínez-García et al. reported infants under five years with a more extensive distribution among all infected groups (23). Meanwhile, Olivar-López et al. described a prevalence in both infants and adolescents as the positive pediatric groups (24). The ages of the infected patients in the studies we analyzed wildly varied, but the discrepancies may derive from differences between the population and location of the studies.

The clinical spectrum of COVID-19 in children is broad. A systematic review and meta-analysis by Qi et al. that included 2,874 patients from 37 articles reported numerous asymptomatic COVID-19 cases, accounting for 27.7% of the sample (28). In another meta-analysis by Behnood et al., the broad rate range of asymptomatic patients was very wide, between 12% and 64% (29). Our study reported 10.1% asymptomatic cases. The variety in the asymptomatic rate might be because its detection is separate from the usual standard, and the protocol for diagnosis may differ among institutions. However, as children present an asymptomatic infection or have less severe symptoms, they are less frequently tested, consequently underestimating the number of infected patients (30). The most common symptoms in order or frequency were fever, cough, and rhinorrhea, just as has been described in other worldwide pediatric cases (2, 14, 17, 23). However, abdominal pain, diarrhea, and vomiting were also found in our study. Gastrointestinal manifestations are commonly reported in young children; in our study, toddlers reported a higher incidence than other age groups (31). A systematic review and meta-analysis of Clinical features of pediatric COVID-19, which included 37 articles and a total of 2,874 patients by Qi et al., described the prevalence of gastrointestinal symptoms between 3.6%–7.2%. They also suggested that despite finding a low prevalence, gastrointestinal manifestation should receive considerable attention when diagnosing pediatric COVID-19, to which we also agreed (28).

Children and adolescents are less likely to develop severe COVID-19 manifestations. Previous studies mentioned that patients with underlying medical conditions are more susceptible to severe illness with higher hospitalization and mortality rates (13, 23, 26, 32). In a cross-sectional study on 43,465 pediatric patients with COVID-19 by Kompaniyets et al., the significant risk factors for hospitalization were type 1 diabetes and obesity. Meanwhile, the risk factors for severe COVID-19 illness were type 1 diabetes and cardiac and circulatory congenital anomalies (33). A Systematic Review and Meta-Analysis by Choi et al. also found obesity and diabetes as risk factors for severe COVID-19 (34). O’Neill et al. reported kidney disease as another risk factor for hospitalization with COVID-19 (32). One of our eight inpatients was already hospitalized for chronic renal failure management when he tested positive for SARS-CoV-2, but his evolution was favorable. Although comorbidities are associated with hospitalization and an unfavorable prognosis, our study could not find such a relation.

Similar to what has been reported, our patients reported non serious clinical manifestations, receiving symptomatic treatment as the only medical management. To date, severe manifestations range between 1%–6% of the cases (13, 15). Of our eight hospitalized patients, only one received Remdesivir as a COVID-19 treatment. Remdesivir is the only antiviral drug approved by the Food and Drug Administration (FDA) for use in hospitalized children with COVID-19 (17, 35). The rest of our inpatients with incidental positive SARS-CoV-2 tests reported an asymptomatic infection and a favorable evolution.

One of the complications of pediatric COVID-19 is MIS-C. A systematic Review of the characteristics associated with COVID-19 in children by Kornitzer et al. reported 543 out of 4,811 (11.28%) with compatible symptoms of MIS-C (36). Other studies describe that children with MIS-C often are hospitalized and require intensive care unit (ICU) management due to possible cardiac complications (35). In our case, only nine (0.6%) patients were diagnosed with MIS-C. None required ICU management; their treatment consisted of immunoglobulin and dexamethasone, as mentioned in the literature (35). To date, none has shown any complications.

The FDA approved the vaccination of adolescents from 12 to 15 years of age in May 2021, and children from 5 to 11 years of age until October 2021 (37, 38). In Mexico, the COVID-19 vaccine was available for adolescents from 15 to 17 years old until December 2021 (39). Nevertheless, by the time this article was written, vaccination campaigns for COVID-19 in Mexico were not available yet. The hospital location is near the frontier with the United States, so the parents had a more promising chance to vaccinate their children against SARS-CoV-2. However, children were required to attend school regardless of their vaccination status. Nearly all the literature on pediatric COVID-19 corresponds to studies that analyze the inpatient/ICU treatments or the disease's behavior in immunocompromised patients. There's few reports of the outpatient clinical characteristics and their evolution along the COVID-19 waves as presented in this study. There is too much to understand about the SARS-CoV-2 infection among individuals with nonserious clinical presentations of COVID-19. This study will undoubtedly contribute to a better understanding of pediatric COVID-19 and the importance of following strict hygiene measures.

We faced several limitations while doing this research. First, the study's retrospective nature stopped us from knowing the authentic time lapse between the onset of the symptoms and testing positive for SARS-CoV-2. Second, parents usually denied or omitted information regarding immunizations received abroad, compromising the vaccination status of the children. Besides, despite not having received a vaccine, children were required to attend school. For future analysis, it could be crucial to confirm this kind of information.

This study aimed to describe the clinical manifestations of the pediatric population with a positive RT-PCR test for SARS-CoV-2 along the four COVID-19 waves in our unit. Most participants were outpatients who reported a mild infection with a favorable evolution based on symptomatic treatment. Despite the majority of our population being unvaccinated or not fully vaccinated, they showed a low rate of complications. Nevertheless, it still needs to be discovered how this will change after schools re-open, as the actual vaccine shortage for children and adolescents in Mexico.

## Data Availability

The datasets presented in this study can be found in online repositories. The names of the repository/repositories and accession number(s) can be found below: https://figshare.com/, https://doi.org/10.6084/m9.figshare.21913479.v1.
